# Folic Acid-Decorated *β*-Cyclodextrin-Based Poly(ε-caprolactone)-dextran Star Polymer with Disulfide Bond-Linker as Theranostic Nanoparticle for Tumor-Targeted MRI and Chemotherapy

**DOI:** 10.3390/pharmaceutics14010052

**Published:** 2021-12-27

**Authors:** Huikang Yang, Nianhua Wang, Ruimeng Yang, Liming Zhang, Xinqing Jiang

**Affiliations:** 1 Department of Radiology, The Second Affiliated Hospital, School of Medicine, South China University of Technology, Yuexiu District, Guangzhou 510640, China; eyyanghk@scut.edu.cn (H.Y.); 201910108741@MAIL.scut.edu.cn (N.W.); eyruimengyang@scut.edu.cn (R.Y.); 2 School of Materials Science and Engineering, Sun Yat-sen University, Haizhu District, Guangzhou 510275, China

**Keywords:** star polymer, *β*-cyclodextrin, tumor-targeted, disulfide bond, theranostic nanoparticles

## Abstract

*β*-cyclodextrin(*β*CD)-based star polymers have attracted much interest because of their unique structures and potential biomedical and biological applications. Herein, a well-defined folic acid (FA)-conjugated and disulfide bond-linked star polymer ((FA-Dex-SS)-*β*CD-(PCL)_14_) was synthesized via a couple reaction between *β*CD-based 14 arms poly(ε-caprolactone) (*β*CD-(PCL)_14_) and disulfide-containing α-alkyne dextran (alkyne-SS-Dex), and acted as theranostic nanoparticles for tumor-targeted MRI and chemotherapy. Theranostic nanoparticles were obtained by loading doxorubicin (DOX), and superparamagnetic iron oxide (SPIO) particles were loaded into the star polymer nanoparticles to obtain ((FA-Dex-SS)-*β*CD-(PCL)_14_@DOX-SPIO) theranostic nanoparticles. In vitro drug release studies showed that approximately 100% of the DOX was released from disulfide bond-linked theranostic nanoparticles within 24 h under a reducing environment in the presence of 10.0 mM GSH. DOX and SPIO could be delivered into HepG2 cells efficiently, owing to the folate receptor-mediated endocytosis process of the nanoparticles and glutathione (GSH), which triggered disulfide-bonds cleaving. Moreover, (FA-Dex-SS)-*β*CD-(PCL)_14_@DOX-SPIO showed strong MRI contrast enhancement properties. In conclusion, folic acid-decorated reduction-sensitive star polymeric nanoparticles are a potential theranostic nanoparticle candidate for tumor-targeted MRI and chemotherapy.

## 1. Introduction

Recently, polymeric nanoparticles have received great attention as theranostic nanoparticles with the ability to deliver drugs and contrasting agents to tumor tissue [1,2,3,4]. Doxorubicin (DOX) is widely used in clinical practice, but it still has some drawbacks that need to be overcome, including poor water solubility, short residence times in circulation, and poor biodistribution. Furthermore, these drugs can cause serious side effects, including nausea, vomiting, cardiotoxicity, myelosuppression, mucositis, hair loss, and systemic toxicity [5,6,7,8]. Nanoparticles were self-assembled from amphiphilic polymers, such as block, grafted, and star polymers in aqueous solution, where the hydrophobic inner core of the nanoparticles can be used to encapsulate a variety of hydrophobic guest molecules [9,10,11]. Polymeric nanoparticles as drug cargo can not only effectively improve the water solubility and pharmacokinetics and prevent the premature burst-release of drugs, but also enhance drug tumor selectivity [12,13].

Currently, there has been increasing interest and attention in incorporating tumor-targeting ligands and stimuli-responsive properties into different nanoparticle systems to create “smart” nanoparticles [14]. Folic acid, galactosyl, lactosyl, Arg-Gly-Asp (RGD) sequence peptode, transferrin, hyaluronic acid, and monoclonal antibodies are widely used as targeting ligands to enhance the tumor-targeting ability of nanoparticles due to their special cell interactions and recognition [9,15,16,17,18,19]. Folic acid as a targeting ligand for tumor drug therapy mainly depends on the presence of the folate receptor on the surface of target cells [20]. Hepatocellular carcinoma is one of the most deadly malignant tumors in the world, and folate receptors (FRs) are overexpressed on hepatocellular carcinoma cells compared with the normal cells [21]. Studies have shown that both free folate and folate-decorated nanoparticles can enter cells via folate receptor-mediated endocytosis [22]. The folic acid-decorated gadolinium-porphyrin metal-organic framework exhibited a strong affinity for folate receptor (FR)-expressing hepatocellular carcinoma cells [23].

Slow drug release from nanoparticles at the tumor site not only compromises therapeutic efficacy but also causes cancer cells to develop multiple-drug resistance (MDR). To address the above limitations, stimuli-responsive “smart” drug carriers, which can release drugs rapidly due to stimulated by external signals at the tumor site, such as redox, pH, enzyme, temperature, light, magnetic force, ultrasound, or voltage signals, have attracted much interest [1,24]. The endogenous reductive agent, glutathione (GSH), is able to cleave disulfide bonds via a redox reaction, and the concentration of GSH is abundant in the cytoplasm of tumor cells [25]. Disulfide bonds are sensitive to GSH; therefore, disulfide bond-linked polymers have been extensively exploited for intracellular drug release [26,27]. Many amphiphilic block copolymers linked with disulfide bonds have been synthesized to act as nanocarriers for intracellular drug delivery and have exhibited higher therapeutic efficacy than reduction-insensitive drug-loaded nanoparticles.

Linear polymers are widely used for drug delivery; however, drug-loaded polymeric nanoparticles are more sensitive to the changes of the block copolymers concentrations, when the concentrations below the critical micelle concentration (CMC) will result in the premature burst release of a drug. Compared with linear polymers, star polymers exhibited some advantages for drug delivery, including their relative insensitivity to the changes of concentration because of their compacted three-dimensional structure, flexible compositions, precise sites for drug conjugation, facile control over chain length, and control of hydrodynamic size and chain end functionality, which can be used to introduce targeting ligands onto the surface of the nanocarrier [28,29,30]. In vivo therapeutic efficacy demonstrated that star polymers drug conjugate exhibited higher antitumor activity and low body weight loss compared to the SN-38 prodrug irinotecan [28]. Tirelli and co-workers found that the degradation rate and drug release kinetics of star-like poly(D,L-lactide) are different from those of linear polymers [31]. Natural *β*-cyclodextrin (*β*CD), which is linked with α-1, 4-glucosidic linkages and 7 α-d-glucose units, and *β*-cyclodextrin-based star polymers have played a significant role in biomedical engineering [32,33,34]. Hassan and co-workers reported the synthesis of biodendrimeric *β*-cyclodextrin as a drug nanocarrier by attaching *β*CD residues to the primary or secondary face of the core *β*CD via a click reaction [35,36]. Seven poly(ester) dendrons were selectively attached to the primary face of *β*CD via a click reaction, and the product as a drug carrier greatly improved the water solubility of albendazole [37]. Shen and co-workers prepared cyclodextrin-centered drug-conjugated amphiphilic star copolymers via the combination of controlled ring-opening polymerization and click reaction. These drug-conjugated star copolymers showed higher drug-loading efficiency than non-drug-conjugated star copolymers [38]. *β*CD-based star polycationics, which were prepared by grafting cationic groups onto the primary or second face of *β*CD, are promising candidates for gene therapy [39,40]. Ai and co-workers prepared an amphiphilic star polysaccharide which, through a click reaction, attached the dextran to the *β-*cyclodextrin core and then modified it with aliphatic chains. After loading SPIO and DOX into the polymeric nanoparticles, the CD-Dex-g-SA/SPIO/DOX theranostic nanoparticles showed great potential for monitoring cancer cells after chemotherapy [41]. 

**Scheme 1:**
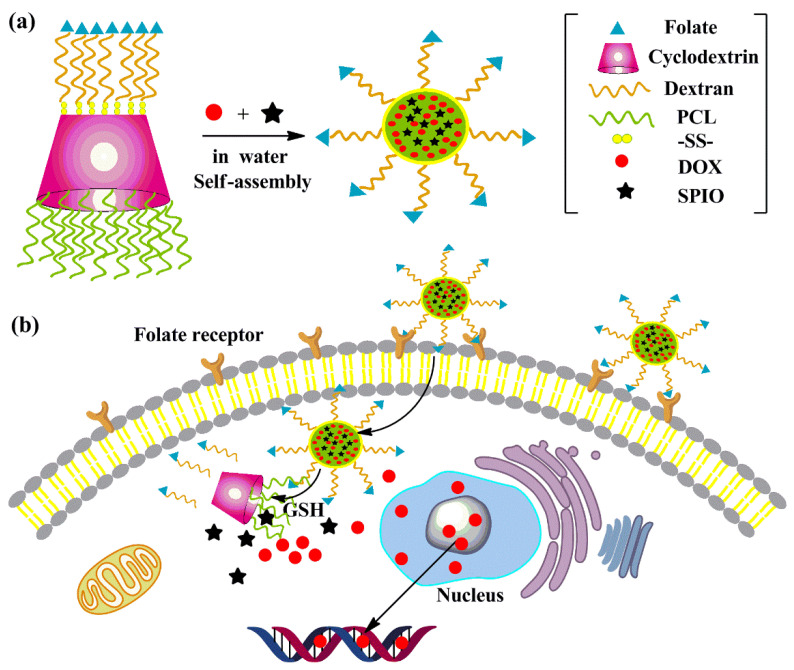
(**a**) Schematic representation of the formation of theranostic nanoparticles, (**b**) folate receptor-mediated cellular uptake and intracellular reduction-controlled drug release for cancer therapy.

In the present research, we report the *β*CD-based star amphiphilic polymers as tumor-targeting redox-sensitive theranostic nanoparticles for tumor therapy and diagnosis (Scheme 1). The star amphiphilic polymers, composed of PCL and folic acid-decorated dextran, were prepared via the combination of ring-opening polymerization of ε-caprolactone with a *β*CD-based initiator ((N_3_)_7_-*β*CD-(OH)_14_) and click conjugation with disulfide bond-linked alkyne terminated dextran (alkyne-SS-dextran) coupled with folic acid. The folic acid groups were expected to increase tumor-targeting capacity, and the introduction of disulfide bonds was expected to accelerate drug release at the tumor sites. The chemical structure was determined by FTIR and ^1^H-NMR. Dynamic light scattering (DLS), transmission electron microscopy (TEM), and fluorescence spectroscopy were applied to study the self-assembly behavior of the *β*CD-based star polymer in aqueous solution. Hydrophobic DOX and SPIO were encapsulated into the hydrophobic inner core by a simple dialysis technique of the nanoparticles. In vitro drug release was carried out in 10-mM GSH to evaluate the effectiveness of the disulfide bonds in controlled drug release. Cellular uptake of the theranostic nanoparticles was studied in the HepG2 cell line (HepG2) by using confocal laser scanning microscopy (CLSM), flow cytometry, and Prussian blue staining. In comparison with counterparts, cell apoptosis and cytotoxicity assays demonstrated that cancer cell growth was markedly inhibited by the tumor-targeting redox-sensitive theranostic nanoparticles. Furthermore, the theranostic nanoparticles showed an excellent MR T_2_ contrast enhancement ability. 

## 2. Materials and Methods

### 2.1. Materials

Folic acid (FA, 99%); tin(II) 2-ethylhexanoate (Sn(Oct)_2_, 95%); ε-caprolactone (ε-CL); N,N,N′,N″,N″-pentamethyldiethylenetriamine (PMDTA); and cuprous bromide (CuBr, 99%) were obtained from Alfa Aesar. *β*CD was purchased from Tokyo Chemical Industry Co., Ltd. (Tokyo, Japan), recrystallized twice from water, and dried under a vacuum drying oven at 50° for 2 days. Doxorubicin hydrochloride (DOX.HCl); sodium cyanoborohydride (NaBH_3_CN, 98%); iodine (I_2_, 98%); triphenylphosphine (TPP, 98%); and glutathione (GSH) were acquired from Aladdin Chemical Company (Shanghai, China). Sodium azide (NaN_3_, 99%) dextran (Mn = 6600 g/mol); propargylamine (98%); and cystamine dihydrochloride (98%) were acquired from Sigma-Aldrich (Saint Louis, MO, USA). Hydroxybenzotriazole (HOBT, 99%) and N-ethyl-N′-(3-dimethylaminopropyl) carbodiimide hydrochloride (EDCI, 98%) were purchased from J&K Chemical Reagent Inc (Beijing, China). Dulbecco’s modified Eagle medium (DMEM), trypsin-ethylenediaminetetraacetic acid (trypsin-EDTA), and fetal bovine serum (FBS) were purchased from Gibco-BRL (Melbourne, Australia). 4′6-Diamidino-2-phenylindole (DAPI) was purchased from Beyotime Institute of Biotechnology (Nanjing, China). 3-(4,5-Dimethylthiazol-2-yl)-2,5-diphe-nyltetrazolium bromide (MTT) was purchased from Invitrogen Corporation (Washington, DC, USA). All other reagents were of analytical grade and used without further purification. The human hepatocellular liver carcinoma (HepG2) cell line was purchased from the Animal Center of Sun Yat-sen University (Guangzhou, China). DMSO-*d*6 and CDCl_3_, used as solvents in the NMR measurements, were purchased from Aldrich. All other reagents were used as received without further purification.

### 2.2. Synthesis of Heptakis(6-deoxy-6-azido)-β-cyclodextrin Centered 14-Arm Star Poly(ε-caprolactone)s ((N_3_)_7_-βCD-(PCL)_14_) via Ring-Opening Polymerization

To obtain the targeted product (N_3_)_7_-*β*CD-(PCL)_14_ via the ring-opening polymerization of ε-CL in Sn(Oct)_2_, the multifunctional initiator, heptakis(6-deoxy-6-azido)-*β*-cyclodextrin, was first prepared, as Liu et al. reported [42]. Typically, 18.36 g of Ph_3_P (70 mmol) was dissolved in anhydrous DMF, and then 17.77 g of I_2_ (70 mmol) was added in 10 min. The Schlenk flask was placed in an oil bath regulated at 70 °C under static nitrogen pressure. Dry *β*-cyclodextrin (5.68 g, 5 mmol) from a vacuum oven was weighed in a glovebox and added to the mixture solution. The black solution was stirred for 24 h at 70 °C under a N_2_ atmosphere. Next, approximately 70 mL of the organic solvent DMF was removed under vacuum. The obtained residue was placed in an ice-water bath, and then 30 mL of sodium methoxide (3.0 M in methanol) was added under stirring. The organic solution was removed under vacuum distillation to acquire black solid residues. The obtained residue was repeatedly refluxed with a Soxhlet extractor, using methanol as the medium for 24 h. After that, the white product heptakis(6-deoxy-6-iodo)-*β*-cyclodextrin ((I_2_)_7_-*β*CD) was collected and dried for 24 h under vacuum at 35 °C. A total of 2.31 g of (I_2_)_7_-*β*CD (1.3 mmol) was weighed and introduced in a Schlenk flask containing 25 mL of anhydrous DMF, and then 1.01 g NaN_3_ (15 mmol) was added. The solution was stirred continuously for 2 days at 70 °C. The organic solvent was removed under reduced pressure and white solid was collected after filtration when the reaction was accomplished. The solid was dried under vacuum at 35 °C for 1 day after being washed with cool water.

A typical synthetic procedure for *β*-cyclodextrin-centered 14-arm star poly(ε-caprolactone)s ((N_3_)_7_-*β*CD-(PCL)_14_) was as follows. First, 0.057 g of (N_3_)_7_-*β*CD (0.6 mmol of OH group), 1.71 g of ε-CL (15 mmol), 0.1 mL of Sn(Oct)_2_ (0.1 mol/L in toluene), and 5.0 mL of dry toluene were added to the flame-dried Schlenk flask with a magnetic stirrer. After the toluene solution and water were removed entirely under vacuum, the argon was exchanged three times with air. The product was vigorously stirred for 24 h in a 110 °C oil bath, the reaction was quenched rapidly by cooling to ambient temperature, and THF was added to the Schlenk flask to dissolve the crude product. Then, the mixture solution was transferred into excessive petroleum ether to collect the precipitate and under vacuum for 24 h until a constant weight was reached (1.67 g, 94.5% overall yield, *M*n = 42,300, *M*w/*M*n = 1.17). ^1^H NMR analysis revealed that the degree of polymerization (DP) for the PCL-OH segments was 23. ^1^H NMR of PCL (500.10 MHz, CDCl_3_, 298 K), *δ*, ppm): 4.1–3.8 (−COOC*H_2_*CH_2_−), 3.6 (−CH_2_C*H_2_*OH), 2.4–2.2 (−CH_2_C*H_2_*COO−), and 1.17–1.12 (−COOCH_2_C*H_2_*C*H_2_*C*H_2_*CH_2_−). FTIR (cm^−^^1^): 1727, 1601, 1489, and 1453. 

### 2.3. Preparation of Disulfide-Containing α-Alkyne Dextran (Alkyne-SS-Dex) by Reductive Amination

As Scheme 1 depicted, the disulfide bonds and the alkyne group were introduced to the dextran end of the molecule simultaneously through reductive amination [43]. Experimentally, 1.2 g of dextran (0.2 mmoL) and propargyl carbamate ethyl dithio ethylamine (PPA-Cyst) were dissolved in DMSO (20 mL), and 0.64 g of NaBH_3_CN was added to the mixture solution as a reducing agent when continuous stirring. Next, 20 mg of NaCNBH_3_ was given every day to sustain the reductive amination at 50 °C for one week. Subsequently, the mixture was transferred into a dialyzed tube (MWCO 1000 Da), and dialysis against acetate buffer (pH = 5.6) for 24 h and deionized water was performed for 48 h. The dialysate was lyophilized to acquire white powder (1.1 g, 87%). 

### 2.4. Synthesis of Star Polymer (Dex-SS)_n_-βCD-(PCL)_14_ by Click Reaction

Click conjugations between alkyne and azido groups are widely used to prepare star polymers [36,44]. Herein, via a click conjugation method between alkyne-SS-Dex and ((N_3_)_7_-*β*CD-(PCL)_14_) to obtain the star polymer, (Dex-SS)_n_-*β*CD-(PCL)_14_ was produced. Briefly, 2.4 g of alkyne-SS-Dex (0.4 mmol of alkyne group) and 0.24 g of (N_3_)_7_-*β*CD-(PCL)_14_ (0.04 mmol of azido group) were added to a round-bottom flask containing 30 mL of anhydrous DMSO under an argon atmosphere, after argon continued bubbling about 15 min and PMDETA was offered. Then, the mixture solution was further bubbled with argon for 15 min after CuBr (30 mg, 0.21 mmol) was introduced. Subsequently, the flask reacted in an oil bath at 50 °C for 3 days. The crude product was transferred into a dialysis tube (MWCO 30,000 Da), and the copper salt was removed by 5% disodium ethylene diate (EDTA-2NA) solution for 2 days, and the excess alkyny-SS-dex was removed by pure water for 3 days. The dialysate was lyophilized to acquire white powder (0.42 g, 87.5%).

### 2.5. Prepare of Folic Acid-Decorated β-Cyclodextrin-Based Poly(ε-caprolactone)-dextran Star Polymer

Folic acid was conjugated onto the star polymer (Dex-SS)_n_-*β*CD-(PCL)_14_ via an esterification reaction between the γ-carboxylic acid group of FA and the hydroxyl group of Dex. Experimentally, 0.20 g of (Dex-SS)_n_-*β*CD-(PCL)_14_ and 0.02 g of folic acid (0.045 mmol) were weighed and dissolved together in 20 mL of anhydrous DMSO, and then EDCI (0.017 g, 0.09 mmol) and HOBT (0.012 g, 0.09 mmol) were added to the solution. The flask was sealed and covered with aluminum foil, and then the mixture solution stirred for 24 h under N_2_ protection. The excess FA of mixture solution was removed by dialysis against deionized water for 3 days. Finally, folic acid-modified *β*-cyclodextrin-based poly(ε-caprolactone)-dextran star polymer ((FA-Dex-SS)_n_-*β*CD-(PCL)_14_) yellow powder was obtained after freeze-drying the dialysate. The degree of substitution (DS) of FA in the synthesized polymer was determined by ^1^H-NMR and calculated as follows:

DS_FA_ = ((*I*_a_ × 441)/2)/(*I*_2−6_)/5 × 162 × 100% (1)

where *I*_a_ is the integral for the protons of poison ring in FA, and *I*_2−6_ is the integral for the protons of *I*_2−6_ in dextran between 3.2 and 4.0 ppm. For comparison, FA-decorated star polymer (FA-Dex)-*β*CD-(PCL)_14_ with reduced insensitivity, star polymer (Dex)-*β*CD-(PCL)_14_ with reduced insensitivity, and FA-decorated star polymer (FA-Dex)-*β*CD-(PCL)_14_ with reduced insensitivity, all with similar compositions, were also prepared.

### 2.6. Characterization

A Perkin-Elmer Paragon1000 spectrometer, with a scanning range from 500 to 4000 cm^−1^, was used to record the Fourier transform infrared (FTIR, Perkin-Elmer Paragon1000 spectrometer, Waltham, MA, USA) spectra by virtue of the potassium bromide (KBr) method at room temperature. The detailed chemical structure of the products was measured on a nuclear magnetic resonance (^1^H NMR, Bruker AV-500, Bruker, Billerrica, MA, USA) spectrometer by deuterated chloroform (CDCl_3_−*d*) or deuterated dimethyl sulfoxide (DMSO−*d*_6_ contain tetramethylsilane (TMS)) as the internal standard, respectively. The number average molecular weight (*M*_n_) and molecular weight polydispersity index (*M*_w_/*M*_n_) of the *β*CD-(PCL)_14_ were recorded by gel permeation chromatography (GPC, Waters 1515, Milford, MA, USA), leading to adoption of a Waters 1515 pump and Waters 2414 differential refractive index detector. The column system was calibrated with a set of monodisperse polystyrene standards using high performance liquid chromatography grade THF as a mobile phase with a flow rate of 0.8 mL/min at 30 °C. A Brookhaven BI-200SM Goniometer particle-size analyzer (Brookhaven Instruments Corporation, NY, USA) was used to determine the hydrodynamic diameter and size distribution of the nanoparticles. The data, collected by an auto-correlator at 25 °C with a detection angle of scattered light at 90°, showed a record lasting 300 s. For each nanoparticle solution, the concentration was fixed at 1 mg/mL and filtered through a 0.45-µm filter. All the data were collected from three measurements. The morphology of the nanoparticles was revealed from a JEOL JEM-2100F transmission electron microscope (TEM, JEM-2100F, JEOL, Tokyo, Japan) which operated at an accelerating voltage of 200 keV. A small drop of micelle solution was suspended on the copper grid surface, before the solution was blotted off with filter paper after 1 min. After that, the sample was negatively stained with 1 wt% phosphotungstic acid solution (2 wt% in water) for 20 s, and the solution was blotted off with filter paper. The grid was completely dried before TEM observation.

### 2.7. Nanoparticles Formation and the Critical Micellar Concentration

Typically, 10 mg of star polymer was dissolved in 2 mL of warm DMSO, and the organic solution was added dropwise into 5 mL of deionized water under vigorous stirring. After the addition of the organic solution, the micelle solution was further stirred for 1 h, and then the mixture solution was placed in a dialysis tube (molecular weight cut-off (MWCO) 3500 Da) and dialyzed against deionized water for 48 h to remove the DMSO. The nanoparticle solution was filtered through a 0.45-µm filter. Then, 2 mL of the nanoparticle solution was lyophilized and weighted, and the nanoparticles concentration was calculated. The final concentration was adjusted to approximately 1 mg/mL.

The critical micellar concentration (CMC) of the final product was determined by the fluorescence probe technique using pyrene as a fluorescence probe [16,45]. Aliquots of pyrene stock solution (6 × 10^−5^ M in acetone, 50 μL) were added to 10-mL Eppendorf tubes, and the acetone was evaporated under vacuum. Then, polymer solutions at different concentrations were added to the Eppendorf tubes, for a final concentration of pyrene of 6 × 10^−7^ M in water. The combined solutions of pyrene and polymer were kept on a shaker at 37 °C for 24 h in the dark to reach the solubilization equilibrium before measurement. The fluorescence excitation spectra were obtained on a fluorescence spectrometer (Shimadzu RF-5301PC fluorescence spectrometer, Kyoto, Japan) at an emission wavelength of 373 nm and a bandwidth of 5 nm, and the scanned range was from 300 to 350 nm at room temperature.

### 2.8. Preparation of DOX and SPIO Co-Loaded Nanoparticles

Then, 10.0 mg of star polymer, 2.0 mg of DOX.HCl, and an equimolar amount of triethylamine (TEA) were co-dissolved in 2 mL of warm DMSO, and then the organic solution was added dropwise to 5 mL of deionized water with vigorous stirring to load the hydrophobic DOX into the inner core of the nanoparticles. The mixture solution was further stirred for 1 h, placed in a dialysis tube (MWCO 3500 Da), and dialyzed against deionized water for 48 h to remove excess DMSO and unloaded DOX. Hydrophobic SPIO (1.5 mg) was dissolved in 1 mL of trichloromethane, and then the organic solution was slowly added into the DOX-loaded nanoparticle solution under sonication. The trichloromethane was removed from the mixture via rotary evaporation. The obtained solution was passed through a 0.22-µm syringe filter to remove the unloaded SPIO precipitate. and the final volume of the solution was adjusted to 10 mL. After dialysis, the UV absorption of the DOX-SPIO-loaded nanoparticles was analyzed by using a UV-vis spectrometer at λ_max_ = 485 nm (Shimadzu UV-3150 UV-vis spectrometer, Kyoto, Japan). Quantification was performed from the calibration curve of DOX in a water-DMSO (*v*/*v* = 30:70) mixture. The DOX-loading content (DLC) was calculated from the followed equation:

*DLC*(%) = *w*_1_/*w*_2_ × 100 (2)

where *w*_1_ is the weight of the loaded drug and *w*_2_ is the weight of the star polymer.

Atomic absorption spectroscopy (AAS, contrAA800, Analytikjena, Jena, Germany) analysis was carried out, and the Fe concentration was determined at the specific Fe absorption wavelength (248.3 nm) based on a previously established calibration curve. The SPIO loading content (SLC) was calculated from the following equation:

*SLC*(%) = *w*_3_/*w*_2_ × 100 (3)

where *w*_3_ is the weight of the loaded SPIO and *w*_2_ is the weight of the star polymer.

### 2.9. In Vitro DOX Cumulative Release from Nanoparticles

In vitro DOX cumulative release from the drug-loaded nanoparticles was investigated in two different media (0 mM or 10 mM GSH) at 37 °C. Specifically, 3 mL of DOX-loaded nanoparticle solution was sealed in a dialysis bag (MWCO 3500 Da) and transferred to 27 mL of PBS containing 0 or 10 mM of GSH with gentle shaking at 100 rpm in a 37 °C water bath. At scheduled time intervals, 3 mL of the external solution was replaced with 3 mL of fresh buffered solution, and DOX release was determined by UV-vis spectrometer (UV-3150, Shimadzu, Japan) at a wavelength of 485 nm [45].

(4) $$E_{r}\left(\%\right)=\frac{V_{e}\sum_{1}^{n-1}C_{i}+V_{0}C_{n}}{m_{\mathrm{DOX}}}\times 100\%$$

where *m*_DOX_ represents the amount of doxorubicin in the nanoparticles, *V*_0_ is the whole volume of the release media (*V*_0_ = 30 mL), *V*_e_ is the volume of the replace media (*V*_e_ = 3 mL), and *C*_i_ represents the concentration of DOX in the *i*th sample.

### 2.10. Cellular Internalization of Nanoparticles Studies

Confocal laser scanning microscopy (Zeiss Elyra P.1 system, Oberkochen, Germany) was exploited to illustrate the interaction between free DOX or DOX-loaded nanoparticles and tumor cells. HepG2 cells were seeded in a 6-well plate at a density of 1 × 10^6^ cells/well in 2 mL of DMEM, and cultured at 37 °C in the 5% CO_2_ atmosphere overnight. Afterwards, the culture medium was replaced with DMEM containing free DOX or DOX-SPIO-loaded nanoparticles (DOX concentration: 10 μg/mL). After 4 h of further incubation, the cells were rinsed twice with PBS and then fixed by 200 μL of 4% paraformaldehyde solution at room temperature for 30 min. Then, the cells were rinsed with PBS three times, DAPI was used to stain cell nuclei for 30 min, and the free dye was washed out with PBS three times. Finally, fluorescence images were captured on a Leica TCS-SP2 confocal laser scanning microscope (Leica, Germany). The excitation wavelength for DOX was 485 nm and that for DAPI was 358 nm. Emission wavelengths were 590 nm for DOX and 455 nm for DAPI.

Flow cytometry was used to quantitatively analyze fluorescence intensity of DOX in HepG2 cells. HepG2 cells were inoculated with 1 × 10^6^ cells/well in a 6-well plate and cultured at 37 °C under 5% CO_2_ atmosphere for 24 h. After removing the culture medium, the cells were incubated with free DOX or loaded DOX-SPIO nanoparticles in DMEM at a concentration of 10 μg/mL for 4 h. Then, the cells were rinsed with PBS and treated with trypsin. Then, 2 mL of complete DMEM was added into a 6-well plate and the cells were collected and resuspended in 0.3 mL of PBS. Cellular uptake of DOX was quantified by flow cytometer (Guava easyCyte 8HT, Merck Millipore, Boston, MA, USA).

Prussian blue staining was performed to detect the cellular uptake of SPIO. In brief, HepG2 cells were inoculated with 1 × 10^6^ cells/well in a 6-well plate and cultured for 24 h at 37 °C under 5% CO_2_ atmosphere, and then the cells were incubated with SPIO-loaded nanoparticles for an additional 4 h. The cells were rinsed out with PBS, and the Prussian blue solution was incubated with cells for 30 min in the dark after being fixed with 4% paraformaldehyde. Finally, HepG2 cells were washed with PBS, and Prussian blue-stained images were collected with an optical microscope (Olympus BX51, Tokyo, Japan).

### 2.11. Cell Cytotoxicity Assays

The in vitro cell cytotoxicity was evaluated on Human hepatocellular liver carcinoma cells using a standard MTT assay. HepG2 cells (1 × 10^4^ cells/well in 100 μL of DMEM) were inoculated into a 96-well plate and incubated 24 h at 37 °C under a 5% CO_2_ atmosphere. The culture medium was replaced with fresh DMEM containing different DOX concentration (0–10 μg/mL), and incubated for 24 or 48 h. After co-incubation with the cancer cells for 24 or 48 h, the previous medium was removed, and then the MTT solution (20 μL/well, 5 mg/mL) was added to the 96-well plate. In order to ensure that MTT could transform into Formazan crystals, the cells were incubated at 37 °C for 4 h. After that, all the supernatants were discarded from the wells and replaced with 200 μL of DMSO, and then the plates were shaken for 10 min to dissolve the formazan crystals. Finally, the absorbance at 490 nm was detected by the microplate reader.

Cell viability (%) = [A_490_ (sample)/A_490_ (control)] × 100 (5)

where A_490_ (sample) and A_490_ (control) are the absorbances of the sample and control wells, respectively.

### 2.12. Cell Apoptosis

The dyes, calcein AM and propidium iodide (PI), were exploited to label apoptotic cells and dead cells. HepG2 cells were seeded into 12-well plates (1 × 10^4^ cells per well) and incubated overnight. Then, the cells were treated with various concentrations (2.5, 5, and 10 μg/mL DOX) free DOX or DOX-SPIO-loaded nanoparticles for 24 h. After that, the cells were collected, washed with PBS two times, and re-suspended in 200 μL of binding buffer. Then, 5 μL of DAPI (E_X_ = 358 nm, E_M_ = 455 nm) solutions and 5 μL of Annexin V-fluorescein isothiocyanate (FITC) (E_X_ = 488 nm, E_M_ = 530 nm) were added to incubate for 15 min on ice to stain dead or apoptotic cells. Finally, the cell apoptosis rates were detected by a Gallios flow cytometer.

### 2.13. Relaxivity Measurement

The *T*_2_ relaxivity of the DOX-SPIO-loaded nanoparticles was recorded on a clinical 3.0 T MRI scanner (Verio, Siemens, Erlangen, Germany) at room temperature, and measurement parameters were used to measure the transverse relaxation time (TR: 1000 ms, TE: 13.8/27.6/41.4/55.2/69.0 ms, flip angle: 180°, slice thickness: 3.0 mm, and matrix: 444 × 448). Relaxation times were obtained by fitting the multi-echo data to a mono-exponential decay curve using linearized least-squares optimization. Relaxivity values were calculated via linear least-squares fitting of 1/relaxation time (s^−1^) versus the iron concentration (mM Fe).

## 3. Results and Discussion

### 3.1. Preparation and Characterization of Folic Acid-Decorated β-Cyclodextrin-Based Poly(ε-caprolactone)-dextran Star Polymer

**Scheme 2:**
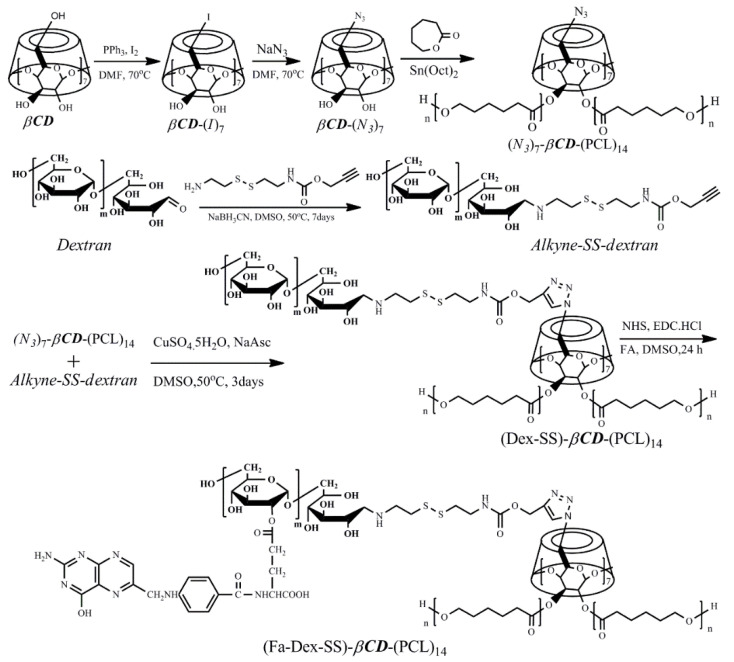
Synthetic route of folic acid-decorated *β*-cyclodextrin-based poly(ε-caprolactone)-dextran star polymer.

**Figure 1:**
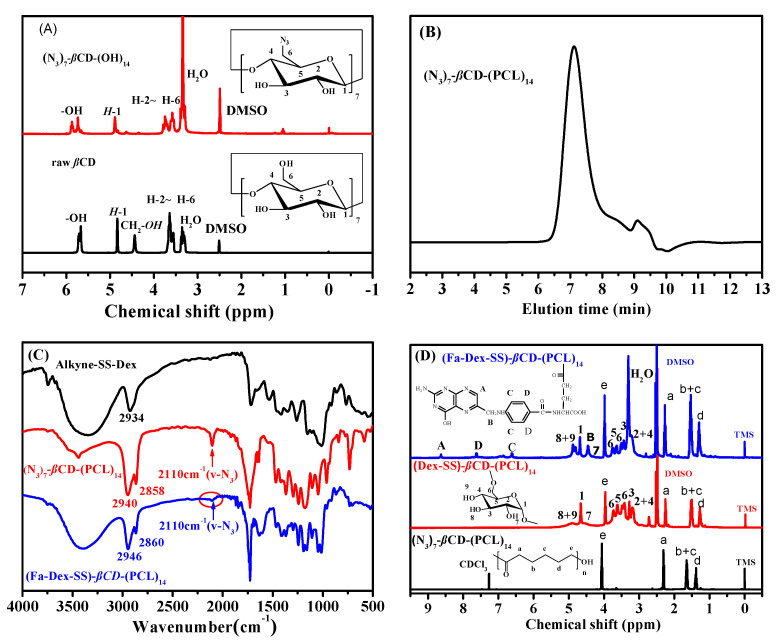
(**A**) ^1^H-NMR spectrum for (N_3_)_7_-*β*CD-(OH)_14_ and raw *β*CD in DMSO-d6; (**B**) THF GPC traces obtained for (N_3_)_7_-*β*CD-(PCL)_14_; (**C**) FTIR spectra for Alkyne-SS-Dex, (N_3_)_7_-*β*CD-(PCL)_14_ and (FA-Dex-SS)-*β*CD-(PCL)_14_; (**D**) ^1^H-NMR spectra for (N_3_)_7_-*β*CD-(PCL)_14_, (Dex-SS)-*β*CD-(PCL)_14_, and (FA-Dex-SS)-*β*CD-(PCL)_14_.

As Scheme 2 illustrates, folic acid-decorated *β*-cyclodextrin-based poly(ε-caprolactone)-dextran star polymer was synthesized by combination of ring opening polymerization (ROP), a copper(I)-catalyzed azide-alkyne cycloaddition reaction, and a standard esterification between dextran and folic acid in the presence of EDCI and HOBt. (N_3_)_7_-*β*CD-(PCL)_14_ was obtained by the following two steps: first, the 7 primary hydroxyl groups of *β*CD were selectively modified into azido groups ((N_3_)_7_-*β*CD-(OH)_14_); and, second, the remaining 14 secondary hydroxyl groups were used as initiators for the ROP of ε-CL in the presence of stannous octoate. The synthesized polymer (N_3_)_7_-*β*CD-(OH)_14_ was characterized by ^1^H NMR. From Figure 1A, we found that the proton signal for 1C-H was located at 4.9 ppm, and the proton signal peaks that appeared between 3.11 to 3.82 belonged to 2C-H~6C-H. The hydroxyl proton signal peaks for 2C and 3C were located at 5.72 and 5.87 ppm, respectively. As compared with the raw *β*CD, the hydroxyl proton signal peak for 6-CH_2_OH at 4.50 ppm completely disappeared, indicating the successful preparation of (N_3_)_7_-*β*CD-(OH)_14_.

Then, (N_3_)_7_-*β*CD-(OH)_14_ was used as a multifunctional initiator, and 14-arm (N_3_)_7_-*β*CD-(PCL)_14_ was synthesized through the ring-opening polymerization of ε-CL in the presence of Sn(Oct)_2_. Then, the mixture was stirred at 110 °C overnight. Figure 1B shows the gel permeation chromatography (GPC) traces of the obtained (N_3_)_7_-*β*CD-(PCL)_14_, and we saw a relatively sharp and symmetrical elution peak. However, the appearance of slight tailing in the low-molecular-weight region was linked to the manifestation of chain transfer side reactions during polymerization. Similar results have been reported by Gou et al. [38]. The average molecular weight was 42,300 g/mol, the weight average molecular weight was 49,500 g/mol, and the molecular weight distribution determined by GPC was 1.17.

The chemical structure of (N_3_)_7_-*β*CD-(PCL)_14_ was further confirmed by FTIR and ^1^H NMR. As Figure 1C showed an absorbance peak at 2110 cm^−1^ in the FTIR spectrum that belonged to the azide groups, the absorption peaks that appeared at 2934 cm^−1^, 2841 cm^−1^ (*ν* C-H), and 1739 cm^−1^ (*ν* C=O) were attributed to the characteristic absorption peaks of PCL chains. As illustrated in Figure 1D, the proton signal peaks belonging to the repeating PCL units were located at 4.1–3.8 ppm (−COOC*H_2_*CH_2_−), 3.6 ppm (−CH_2_C*H_2_*OH), 2.4–2.2 ppm (−CH_2_C*H_2_*COO−), and 1.17–1.12 ppm (−COOCH_2_C*H_2_*C*H_2_*C*H_2_*CH_2_−). Furthermore, the degree of polymerization (DP) of (N_3_)_7_-*β*CD-(PCL)_14_ was approximately 23, which was calculated by comparing the integration area attributed to protons of the PCL between 4.1–3.8 ppm (−COOC*H_2_*CH_2_−) to the signals that appeared at 3.65 ppm (−COOCH_2_CH_2_CH_2_CH_2_C*H*_2_OH). From the above results, we could confirm that the 14-arm (N_3_)_7_-*β*CD-(PCL)_14_ polymer was successfully synthesized.

Many functional groups, such as alkyne or azide groups, can be introduced onto the reducing end of polysaccharides through a reductive amination reaction. Herein, α-alkyne-SS-dextran was synthesized using a procedure similar to that reported by Lecommandoux for the preparation of α-alkyne-dextran [43]. From the ^1^H NMR analysis, we found that the proton peaks belonging to the reducing end group, i.e., the anomeric proton (α-centred at 6.7 ppm and *β*-centred at 6.3 ppm), entirely disappeared, which signified compelling evidence of quantitative reaction. 

In this work, a grafting-onto strategy was applied to synthesize *β*-cyclodextrin-based poly(ε-caprolactone)-dextran star polymer between (N_3_)_7_-*β*CD-(PCL)_14_ and α-alkyne-SS-dextran in DMSO solution at 60 °C for 48 h in the presence of CuBr/PMDETA. In order to synthesize a well-defined (Dex-SS)-*β*CD-(PCL)_14_ star polymer, the molar ratio of α-alkyne-SS-dextran to (N_3_)_7_-*β*CD-(PCL)_14_ was 10: 1. After click reaction, excess α-alkyne-SS-dextran precursor was facilely removed via dialysis against deionized water for 3 days in a dialysis tube (MWCO 30,000 Da). From the ^1^H NMR spectrum of (Dex-SS)-*β*CD-(PCL)_14_ (Figure 1D), the signals at 4.1–3.8, 3.6, 2.4–2.2, and 1.17–1.12 ppm were due to the methylene protons of the PCL arms, and the dextran proton signals were located at 3.1–4.0 (m, dextran glucosidic protons), 4.7 (s, dextran anomeric proton), 4.5, 4.9, and 5.1 ppm (s, dextran hydroxyl protons), indicating the formation of a (Dex-SS)-*β*CD-(PCL)_14_ star polymer. However, as Figure 1C indicated, a weak azide group characteristic absorption peak was still observed at 2094 cm^-1^ in the FTIR spectrum for the (Dex-SS)-*β*CD-(PCL)_14_. This result confirmed that some azide groups remained, even though the α-alkyne-SS-dextran/(N_3_)_7_-*β*CD-(PCL)_14_ molar feed ratio was 10: 1. A similar result was reported by Ai et al., who tried to graft α-alkyne-dextran onto (N_3_)_7_-*β*CD, and found that the steric hindrance of dextran could directly limit the coupling efficiency. Even when the alkyne/azide molar ration reached up to 14:1, more than two azide groups remained unreacted [41].

(FA-Dex-SS)-*β*CD-(PCL)_14_ star polymer was prepared via an esterification reaction between the γ-carboxylic acid group of FA and the hydroxyl group of dextran. The chemical structure of the targeting compound was confirmed by ^1^H NMR. As demonstrated in Figure 1D, by comparison with the ^1^H NMR spectrum of (Dex-SS)-*β*CD-(PCL)_14_, we find that three new proton signal peaks appeared at 8.6, 7.7, and 6.8 ppm, which were attributed to the pyrazine and aromatic proton signals of folic acid. Moreover, the degree of substitution (DS) of FA in (FA-Dex-SS)-*β*CD-(PCL)_14_ was determined to be 11 wt% (approximately 1.7 FA molecules per dextran), according to the proton peak integrations of the aromatic of FA and dextran protons between 3.2 and 4.0 ppm [46].

### 3.2. Preparation and Characterization of Nanoparticles

**Figure 2:**
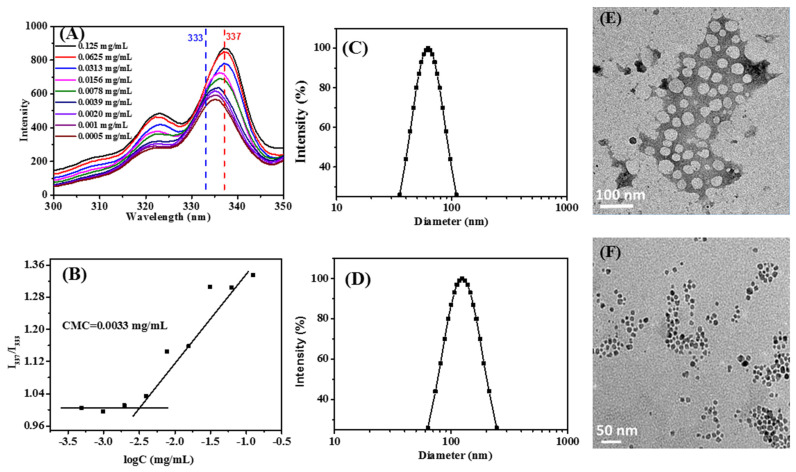
(**A**) Excitation spectrum for pyrene in (FA-Dex-SS)-*β*CD-(PCL)_14_ aqueous solutions, (**B**) plots of fluorescence intensity ratio *I*_337_/*I*_333_ from pyrene excitation spectra vs. log concentration of (FA-Dex-SS)-*β*CD-(PCL)_14,_ (**C**) size distribution for blank (FA-Dex-SS)-*β*CD-(PCL)_14_ solution (1 mg/mL) at 90° and 20 °C, (**D**) size distribution for DOX-SPIO loading (FA-Dex-SS)-*β*CD-(PCL)_14_ solution (1 mg/mL) at 90° and 20 °C, (**E**) TEM micrography of blank (FA-Dex-SS)-*β*CD-(PCL)_14_ nanoparticles, and (**F**) TEM micrography of DOX-SPIO-loading (FA-Dex-SS)-*β*CD-(PCL)_14_ nanoparticles.

The (FA-Dex-SS)-*β*CD-(PCL)_14_ star polymer is composed of hydrophilic dextran arms and hydrophobic PCL arms; therefore, this amphiphilic star polymer can form aggregates in aqueous medium. The critical micelle concentration (CMC) value is an important parameter which was detected by fluorescence of pyrene to describe the formation of aggregates. Figure 2A showed the fluorescence excitation spectra of pyrene in (FA-Dex-SS)-*β*CD-(PCL)_14_ aqueous solutions at various concentrations. At lower concentrations, the fluorescence intensity of pyrene was weak, and the peak nearly unchanged at 335 nm, indicating that pyrene was still in a polar environment. As the star polymer concentrations increased, the fluorescence intensity of pyrene dramatically increased, and an obvious redshift of the excitation peak was observed. It has been reported that pyrene tends to show strong fluorescence intensity when it is encapsulated into hydrophobic core; hence, fluorescence excitation spectra confirmed that pyrene was incorporated into the hydrophobic inner of the nanoparticles [47]. By calculating the ratios of pyrene fluorescence intensities (*I*_337_/*I*_333_) and the concentration of (Fa-Dex-SS)-*β*CD-(PCL)_14_, the CMC value was approximately 0.0033 mg/mL, which is much lower than 0.2 mg/mL for D-a-tocopheryl polyethylene glycol succinate 2000 (Vitamin E TPGS2k) micelles [48]. The lower CMC means that the (Fa-Dex-SS)-*β*CD-(PCL)_14_ nanoparticles would provide good stability for the drug delivery during the blood circulation.

(FA-Dex-SS)-*β*CD-(PCL)_14_-based blank or drug-loaded nanoparticles were prepared using the nanoprecipitation method, and the particle size and morphology of these nanoparticles were measured by DLS and TEM at a constant concentration of 1 mg/mL. Particle size and distribution were recorded at a constant scattering angle of 90°. As shown in Figure 2C, a monodispersed size distribution was found, the particle size of the blank nanoparticles was approximately 65 nm, and the polydispersity index was 0.12 in aqueous solution. The morphology of the blank nanoparticles is illustrated in Figure 2E, while TEM imaging showed the formation of well-defined spherical particles. It should be noticed that the particle size of the blank nanoparticles measured by DLS is larger than found from the TEM results, which was attributed to the fact that DLS results were recorded in aqueous solution, while TEM measures the actual size of the nanoparticles.

As illustrated in Figure 2D and Table 1, the particle size of the theranostic nanoparticles measured by DLS was larger than blank nanoparticles. The increase in particle size could be attributed to the encapsulation of hydrophobic DOX and the MR contrasting agent SPIO into the hydrophobic core of the (FA-Dex-SS)-*β*CD-(PCL)_14_ nanoparticles [49]. These SPIO nanoparticles were well-defined spheroids, as shown in Figure 2F, and were observed by TEM. In addition, the DLC and SLC of the star-like polymeric nanoparticles are shown in Table 1. The calculated DOX-loading content in the star-like polymeric nanoparticles was approximately 11.0%, and the SPIO loading content was about 10.0%.

### 3.3. Reduction Triggered Drug Release

**Figure 3:**
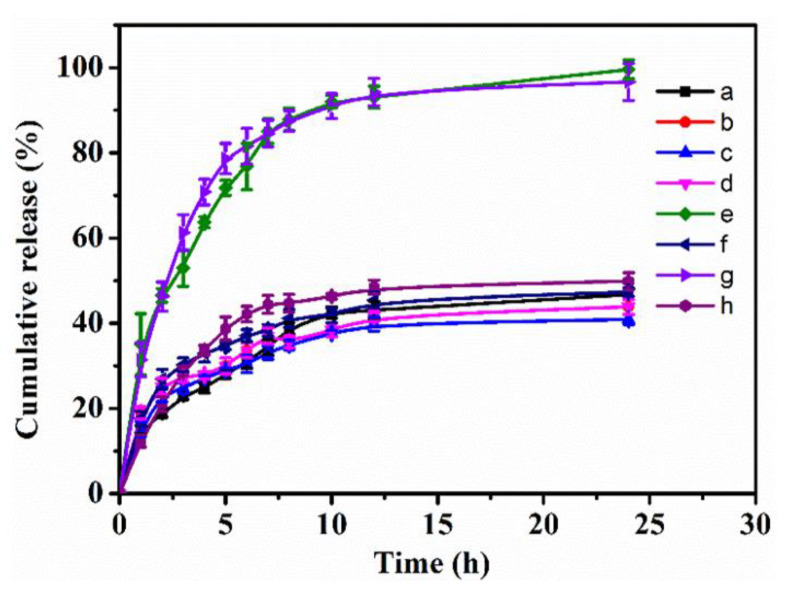
In vitro release of DOX from (a) (FA-Dex-SS)-*β*CD-(PCL)_14_, (b) (FA-Dex)-*β*CD-(PCL)_14_, (c) (Dex-SS)-*β*CD-(PCL)_14_ and (d) (Dex)-*β*CD-(PCL)_14_ nanoparticles incubated without GSH; DOX release from loaded DOX (e) (FA-Dex-SS)-*β*CD-(PCL)_14_, (f) (FA-Dex)-*β*CD-(PCL)_14_, (g) (Dex-SS)-*β*CD-(PCL)_14_, and (h) (Dex)-*β*CD-(PCL)_14_ nanoparticles incubated with 10-mM GSH. The data were presented as mean ± SD (*n* = 3).

Reduction-sensitive drug delivery systems can release drugs faster in a highly reductive tumor microenvironment, where the disulfide bond can be cleaved in the presence of GSH. For instance, Zhong et al. prepared disulfide bond-linked dextran-SS-PCL liner block polymers and applied them for the reduction-triggered release of DOX [50]. Herein, the DOX release behavior from the star polymer nanoparticles was studied in PBS with different GSH concentrations (0 mM, 10 mM) at 37 °C. Figure 3 shows the cumulative release percentages of loaded DOX in the star polymer nanoparticles versus time. In the case of 0-mM GSH, analogous to normal physiological conditions, the release of DOX from the star polymer nanoparticles was slow, and approximately 46.74, 43.86, 40.89, and 43.86% of the loaded DOX was released from (FA-Dex-SS)-*β*CD-(PCL)_14_, (FA-Dex)-*β*CD-(PCL)_14_, (Dex-SS)-*β*CD-(PCL)_14_, and (Dex)-*β*CD-(PCL)_14_ nanoparticles, respectively, within 24 h. In the case of 10-mM GSH, only approximately 47.31 and 49.8% of DOX was released from DOX-loaded (FA-Dex)-*β*CD-(PCL)_14_ and (Dex)-*β*CD-(PCL)_14_ nanoparticles, respectively, within 24 h. These results indicated that the presence of GSH has no effect on the release of DOX from reduction-insensitive nanoparticles, and the release of DOX was sustained. However, fast release of DOX was observed from the disulfide bond-linked (FA-Dex-SS)-*β*CD-(PCL)_14_ and (Dex-SS)-*β*CD-(PCL)_14_ nanoparticles in the case of 10-mM GSH, which mimicked the intracellular environment, and the DOX cumulative release reached nearly 70% within the first 5 h and approximately 100% within 24 h. Similarly, Park et al. found that CPT was completely released from reduction-sensitive nanoparticles within 20 h, which was caused by cleavage of the disulfide bond in the PEG-SS-PBLG copolymer [27]. The DOX rapidly released from the disulfide bond-linked nanoparticles in this experiment could ascribe to the cleavage of the disulfide bonds by GSH [10,26]. 

### 3.4. In Vitro Cell Cellular Uptake and Cytotoxicity

**Figure 4:**
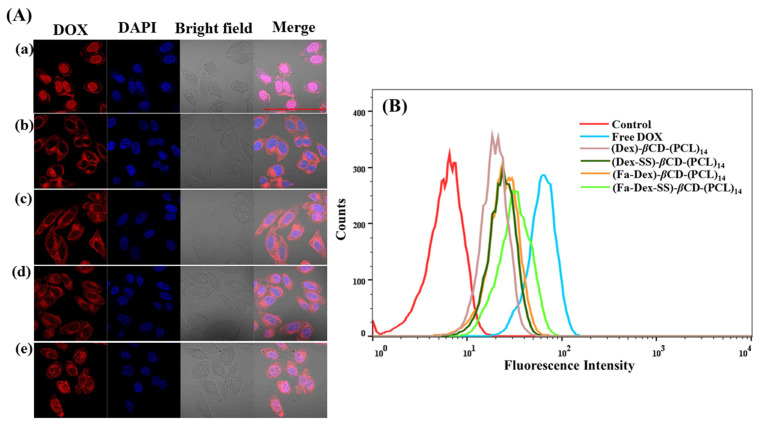
In vitro cell cellular uptake analysis by (**A**) CLSM after HepG2 cells incubated with (**a**) Free DOX, (**b**) (Dex)-*β*CD-(PCL)_14_@DOX-SPIO, (**c**) (Dex-SS)-*β*CD-(PCL)_14_@DOX-SPIO, (**d**) (FA-Dex)-*β*CD-(PCL)_14_@DOX-SPIO and (**e**) (FA-Dex-SS)-*β*CD-(PCL)_14_@DOX-SPIO at 5 μg/mL DOX for 4 h (scale bar = 100 μm). (**B**) DOX fluorescence intensity of HepG2 cells incubated with free DOX and DOX-SPIO-loaded polymeric nanoparticles at 5 μg/mL of DOX for 4 h measured by flow cytometry.

It is well known that the anticancer effects of DOX are due to DOX bounding to chromosomal DNA, leading to cell death [51,52]. Therefore, the internalization and subcellular distribution of DOX play an important role in the anticancer effects. Herein, CLSM was performed to evaluate the effects of FA and disulfide bonds on the cellular uptake behavior and intracellular distribution of DOX in folate receptor (FR)-positive HepG2 cells. CLSM images in Figure 4A show that HepG2 cells treated with free DOX exhibited stronger intracellular DOX fluorescence intensity than cells incubated with DOX-SPIO-co-loaded star polymeric nanoparticles in 4 h, whereas free DOX accumulated in the nucleus predominately. This result can be attributed to the DOX concentration gradient in which DOX passively diffused into the cytosol and was then taken into the nucleus to intercalate into chromosomal DNA [53]. In comparison, the intracellular DOX fluorescence of HepG2 cells incubated with (Dex)-βCD-(PCL)14@DOX-SPIO nanoparticles was notably present in the cytoplasm, with almost no fluorescence in the nucleus, while DOX fluorescence of (Dex-SS)-βCD-(PCL)14@DOX-SPIO nanoparticles was prominently accumulated in the cytosol and substantially less accumulated in the nuclei. This consequence could be attributed to the accelerated DOX release from the nanoparticles under reducing conditions, while DOX diffused into the nuclei [50]. In HepG2 cells, incubated with the FA-decorated (FA-Dex)-*β*CD-(PCL)_14_@DOX-SPIO nanoparticles, strong DOX fluorescence accumulated in the cytosol, and weak fluorescence accumulated in the nuclei. This result could be associated with folate receptor-mediated endocytosis mechanism. Gong and co-workers found that FA-conjugated SPIO-DOX-loaded vesicle-treated HeLa cells showed higher DOX fluorescence than cells treated with FA-free vesicles [54]. Notably, strong DOX fluorescence was observed in the cytoplasm and the nuclei when HepG2 cells were incubated with FA-conjugated reduction-sensitive nanoparticles (FA-Dex-SS)-*β*CD-(PCL)_14_@DOX-SPIO 4 h. These results not only demonstrated that DOX could be efficiently transported into the cytoplasm but also suggested that DOX was rapidly released from the nanoparticles diffused into the cell nuclei when under reducing conditions.

Flow cytometric analysis was performed to further quantify intracellular DOX uptake by HepG2 cells. The fluorescence intensity of DOX in HepG2 cells was quantitatively assayed after free DOX or DOX-SPIO-loaded star polymeric nanoparticles at a DOX concentration of 5 μg/mL cultured for 4 h. As illustrated in Figure 4B, cells cultured with free DOX showed higher fluorescence intensity, owing to a diffusion mechanism of DOX cellular uptake, while DOX-SPIO-loaded star polymeric nanoparticles cultured cells showed lower fluorescence intensity, dependent on endocytosis. Flow cytometric analysis demonstrated that the cells treated with nanoparticles, decorated with an FA-targeting ligand or with a disulfide bonds linkage, exhibited higher DOX fluorescence intensity than FA- and disulfide bond-free nanoparticles. The above results confirmed that the folate receptor-mediated endocytosis process or reduction-responsive drug release behavior can enhance the intracellular DOX fluorescence intensity. Furthermore, we wondered whether the introduction of folic acid and disulfide bonds contemporary could more efficiently enhance intracellular DOX uptake. To confirm this, the FA-targeting ligands and disulfide bond star polymer (FA-Dex-SS)-βCD-(PCL)14@DOX-SPIO was developed as a theranostic nanoparticle for tumor treatment and diagnosis. As anticipated, cells which were treated with (FA-Dex-SS)-βCD-(PCL)14@DOX-SPIO nanoparticles exhibited the strongest intracellular fluorescence of DOX among all these cells treated by star polymeric nanoparticles, which suggests that cellular uptake of these nanoparticles was rapid, as was the release of DOX from nanoparticles. These results imply that the (FA-Dex-SS)-*β*CD-(PCL)_14_ polymer is a promising nano-cargo for tumor-targeted drug delivery, which has conformance with the CLSM observations. 

**Figure 5:**
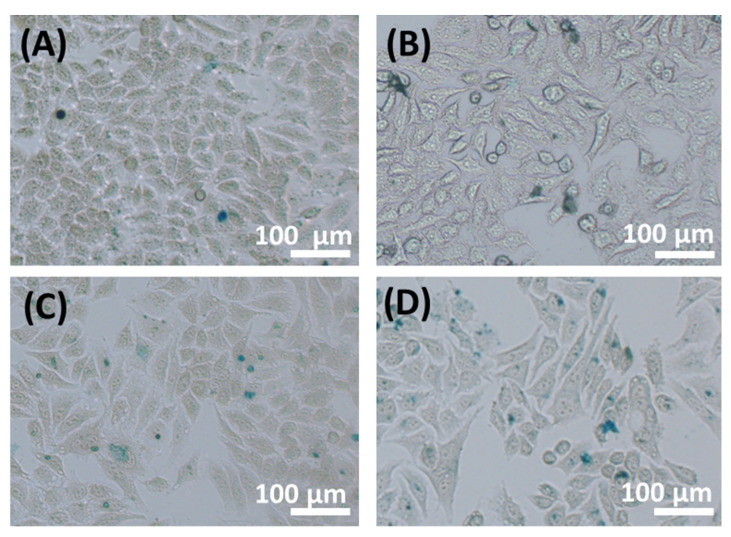
Prussian blue staining images of HepG2 cells incubated with (**A**) (Dex)-*β*CD-(PCL)_14_@DOX-SPIO, (**B**) (Dex-SS)-*β*CD-(PCL)_14_@DOX-SPIO, (**C**) (FA-Dex)-*β*CD-(PCL)_14_@DOX-SPIO and (**D**) (FA-Dex-SS)-*β*CD-(PCL)_14_@DOX-SPIO at 5 μg/mL SPIO for 4 h.

In vitro cellular uptake effects of the star polymeric nanoparticles were demonstrated by Prussian blue staining. As shown in Figure 5, blue spots appeared when HepG2 cells were treated with DOX-SPIO-loaded star polymeric nanoparticles for 4 h. Similar to the CLSM and flow cytometric results, the cells incubated with (FA-Dex-SS)-*β*CD-(PCL)_14_@DOX-SPIO nanoparticles showed more blue spots than their counterparts.

**Figure 6:**
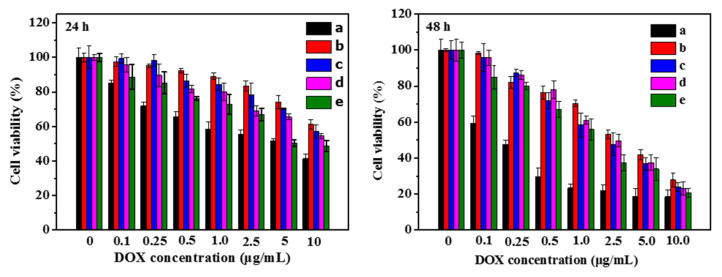
In vitro cell cytotoxicity of HepG2 cells incubated with (a) free DOX, (b) (Dex)-*β*CD-(PCL)_14_@ DOX-SPIO, (c) (Dex-SS)-*β*CD-(PCL)_14_@ DOX-SPIO, (d) (FA-Dex)-*β*CD-(PCL)_14_@ DOX-SPIO, and (e) (FA-Dex-SS)-*β*CD-(PCL)_14_@ DOX-SPIO for 24 and 48 h. The error bars in the graph represent standard deviations (*n* = 3).

The MTT assay was applied to evaluate in vitro cytotoxicity of free DOX and DOX-SPIO-loaded *β*CD base star polymeric nanoparticles towards HepG2 cells. MTT assay results in Figure 6 showed that cell growth inhibition was limited by DOX concentrations and incubation time. Normally, free DOX exhibits higher cytotoxicity to HepG2 cells than DOX-SPIO-loaded star polymeric nanoparticles, due to free DOX transported into cells through a passive diffusion mechanism. Notably, after 48 h of incubation, the FA-conjugated ((FA-Dex)-*β*CD-(PCL)_14_) or less sensitive ((Dex-SS)-*β*CD-(PCL)_14_) DOX-SPIO-loaded polymeric nanoparticles showed higher cytotoxicity than the polymeric nanoparticles without targeting molecules or disulfide bonds. It is worth paying more attention to the efficacy of tumor cell growth inhibition for (FA-Dex-SS)-*β*CD-(PCL)_14_@DOX-SPIO nanoparticles, which was markedly enhanced compared to (Dex)-*β*CD-(PCL)_14_@DOX-SPIO and (Dex-SS)-*β*CD-(PCL)_14_@DOX-SPIO nanoparticles. This phenomenon was probably caused by folate receptor-mediated internalization and cleavage of the disulfide bonds in the reducing environment.

**Figure 7:**
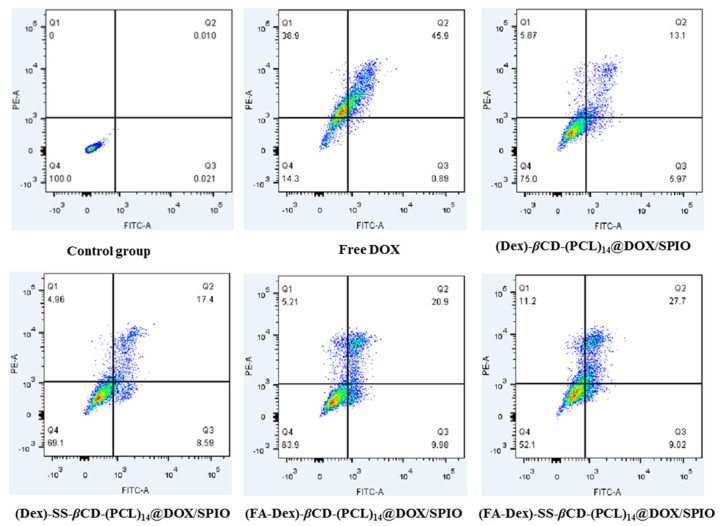
Cells apoptosis analysis by flow cytometry after HepG2 cells incubated with free DOX and DOX-loaded nanoparticles at 5 μg/mL of DOX for 24 h.

The quantitative determination of the apoptotic and necrotic activities of free DOX and DOX-SPIO-loaded polymeric nanoparticles towards HepG2 cells was further investigated by Annexin V-FITC and DAPI flow cytometric assays at a DOX dose of 5 μg/mL. As illustrated in Figure 7, cells incubated in pure culture medium for 24 h showed very low apoptotic activity. HepG2 cells treated with free DOX demonstrated the strongest effect on cell apoptosis (46.79%) among all the test groups, which was attributed to the passive diffusion cellular uptake mechanisms. As a reduction-insensitive nanocarrier without targeting capacity, the ratio of apoptotic cells in the (Dex)-*β*CD-(PCL)_14_@DOX-SPIO group was approximately 19.07%. In comparison, DOX delivered by reduction-sensitive (Dex-SS)-*β*CD-(PCL)_14_ polymeric nanoparticles resulted in 25.99% cell apoptosis. A better apoptosis effect resulted from the cells incubated with FA-decorated (FA-Dex)-*β*CD-(PCL)_14_@DOX-SPIO nanoparticles, leading to 30.88% apoptotic cells. As expected, cells treated with (FA-Dex-SS)-βCD-(PCL)14@DOX-SPIO polymeric nanoparticles performed excellently on leading cells, with a percentage of apoptotic cells reaching up to 36.72%. The above results demonstrated that the *β*-cyclodextrin-based poly(ε-caprolactone)-dextran star polymer with FA conjugation and reduction-triggered properties showed promising potential as a tumor-targeted drug nanocarrier.

### 3.5. MRI Contrast Measurement

**Figure 8:**
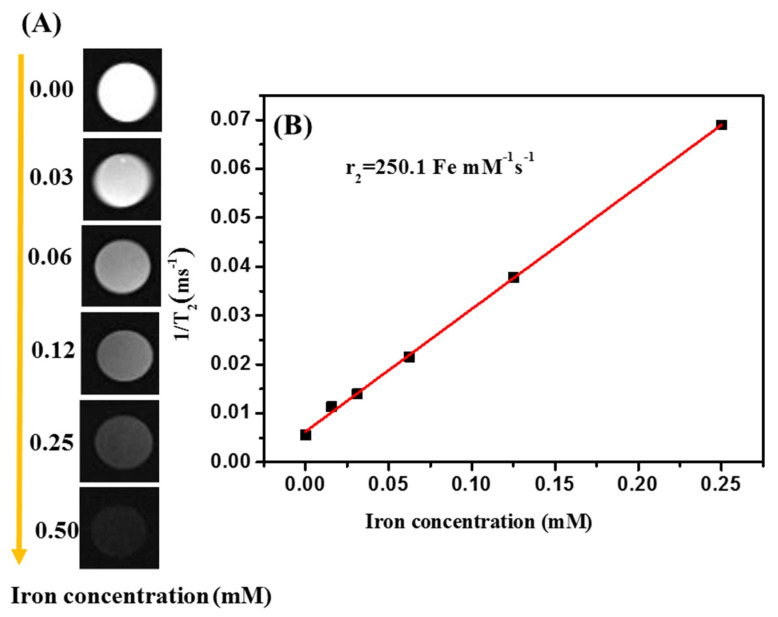
(**A**) *T*_2_-mapping images and (**B**) *T*_2_ relaxation rate of (FA-Dex-SS)-*β*CD-(PCL)_14_@DOX-SPIO nanoparticles at various Fe concentrations (mM).

Currently, the development of SPIO-based theranostic nanoparticles has attracted considerable attention, and SPIO-based MRI contrast agents can dramatically shorten the *T*_2_ (spin-spin) relaxation times [55]. *T*_2_ relaxivity can be calculated from the linear relationship of 1/relaxation time (s^−1^) versus iron concentrations (mM Fe). In this work, the *T*_2_ relaxation rate of (FA-Dex-SS)-*β*CD-(PCL)_14_@DOX-SPIO nanoparticles was measured in a clinical MRI scanner with a 3.0-T field strength. The negative contrast enhancement ability of (FA-Dex-SS)-*β*CD-(PCL)_14_@DOX-SPIO nanoparticles was evaluated from the *T*_2_-weighted images in Figure 8A. As the *T*_2_-weighted images illustrated, as the iron concentration increased, the signals darkened dramatically. Figure 8B shows the *T*_2_ rates of the SPIO-loaded nanoparticles versus iron concentrations, and the transverse relaxation time calculated from the *T*_2_ rate of the iron concentration was approximately 250.1 Fe mM^−1^s^−1^, indicating an improved relaxation property. It has been reported that SPIO nanoparticles can be loaded into the compact inner core of nanoparticles, which can shorten the distance of each SPIO and favor magnetic coupling between the SPIO, increasing *r*_2_ [41,56].

## 4. Conclusions

In this study, a folic acid-decorated *β*-cyclodextrin-based poly(ε-caprolactone)-dextran star polymer was successfully synthesized via the ring opening polymerization combined with Cu(I) catalyed click reaction and performed as a theranostic nanoparticle for tumor-targeted MRI and chemotherapy. The chemical characteristics of the star polymer were confirmed by GPC, FTIR, and ^1^H NMR analyses. Due to this, the amphiphilic features of star polymer could self-assemble into spherical nanoparticles with an average size of approximately 70 nm. Theranostic nanoparticles were obtained after loading DOX and SPIO inside the core of these nanoparticles. In vitro drug release experiments confirmed that disulfide bond-linked theranostic nanoparticles rapidly released DOX in the presence of a high GSH concentration, due to the cleavage of the disulfide bonds would be more favorable at the higher GSH concentration. In vitro cellular uptake and cytotoxicity assays suggested that FA-decorated reduction-sensitive nanoparticles exhibited higher intracellular uptake and a greater proliferation inhibitory effect than their counterparts, possibly because the micelle was taken up by the HepG2 cells through a folate receptor-mediated endocytosis process, and the DOX was readily released under high GSH level intracellular environment. Moreover, after loading SPIO inside the compact core of the nanoparticles, the *T*_2_ relaxation rate increased to 250.1 Fe mM^−1^s^−1^. Therefore, FA-decorated reduction-sensitive star polymeric nanoparticles show great potential as theranostic nanocarriers for tumor-targeted drug delivery and diagnosis applications.

## Figures and Tables

**Table 1:** Characteristics of blank and DOX-SPIO-loaded nanoparticles.

Sample Code	Blank Nanoparticles	DOX-SPIO-Loaded Nanoparticles		
	Diameter *^a^*	Diameter (nm) *^a^*	DLC (%)	SLC (%)
(FA-Dex-SS)-*β*CD-(PCL)_14_	65.8 ± 2.3	126.8 ± 2.3	11.7	10.3
(FA-Dex)-*β*CD-(PCL)_14_	77.8 ± 2.3	124.4 ± 1.7	11.2	10.5
(Dex-SS)-*β*CD-(PCL)_14_ (Dex)-*β*CD-(PCL)_14_	72.3 ± 1.5 86.7 ± 3.5	127.8 ± 3.7 155.8 ± 1.9	10.3 10.8	10.1 9.8

All aggregate solutions had a final polymer concentration of 0.50 mg/mL. *^a^* Measured by dynamic light scattering (DLS).

## Data Availability

Due to privacy or ethical restrictions, the data that support the findings of this study are not publicly available but are available on request from the corresponding author.

## References

[1] Zhou L., Wang H., Li Y. (2018). Stimuli-Responsive Nanomedicines for Overcoming Cancer Multidrug Resistance. Theranostics.

[2] Fan C.H., Cheng Y.H., Ting C.Y., Ho Y.J., Hsu P.H., Liu H.L., Yeh C.K. (2016). Ultrasound/Magnetic Targeting with SPIO-DOX-Microbubble Complex for Image-Guided Drug Delivery in Brain Tumors. Theranostics.

[3] Yilmaz G., Demir B., Timur S., Becer C.R. (2016). Poly(methacrylic acid)-Coated Gold Nanoparticles: Functional Platforms for Theranostic Applications. Biomacromolecules.

[4] Janib S.M., Moses A.S., MacKay J.A. (2010). Imaging and drug delivery using theranostic nanoparticles. Adv. Drug Deliv. Rev..

[5] Hu M., Shen Y., Zhang L., Qiu L. (2016). Polymersomes via Self-Assembly of Amphiphilic β-Cyclodextrin-Centered Triarm Star Polymers for Enhanced Oral Bioavailability of Water-Soluble Chemotherapeutics. Biomacromolecules.

[6] De Beer E.L., Bottone A.E., Voest E.E. (2001). Doxorubicin and mechanical performance of cardiac trabeculae after acute and chronic treatment: A review. Eur. J. Pharmacol..

[7] Kim J.E., Cho H.J., Kim J.S., Shim C.K., Chung S.J., Oak M.H., Yoon I.S., Kim D.D. (2013). The limited intestinal absorption via paracellular pathway is responsible for the low oral bioavailability of doxorubicin. Xenobiotica.

[8] Turakhia S., Venkatakrishnan C.D., Dunsmore K., Wong H., Kuppusamy P., Zweier J.L., Ilangovan G. (2007). Doxorubicin-induced cardiotoxicity: Direct correlation of cardiac fibroblast and H9c2 cell survival and aconitase activity with heat shock protein 27. Am. J. Physiol. Heart Circ. Physiol..

[9] Upadhyay K.K., Bhatt A.N., Mishra A.K., Dwarakanath B.S., Jain S., Schatz C., Le Meins J.F., Farooque A., Chandraiah G., Jain A.K. (2010). The intracellular drug delivery and anti tumor activity of doxorubicin loaded poly(gamma-benzyl L-glutamate)-b-hyaluronan polymersomes. Biomaterials.

[10] Yang H.-K., Qi M., Mo L., Yang R.-M., Xu X.-D., Bao J.-F., Tang W.-J., Lin J.-T., Zhang L.-M., Jiang X.-Q. (2016). Reduction-sensitive amphiphilic dextran derivatives as theranostic nanocarriers for chemotherapy and MR imaging. RSC Adv..

[11] Tang H., Zhang J., Tang J., Shen Y., Guo W., Zhou M., Wang R., Jiang N., Gan Z., Yu Q. (2018). Tumor Specific and Renal Excretable Star-like Triblock Polymer-Doxorubicin Conjugates for Safe and Efficient Anticancer Therapy. Biomacromolecules.

[12] Jain R.K., Stylianopoulos T. (2010). Delivering nanomedicine to solid tumors. Nat. Rev. Clin. Oncol..

[13] Wicki A., Witzigmann D., Balasubramanian V., Huwyler J. (2015). Nanomedicine in cancer therapy: Challenges, opportunities, and clinical applications. J. Control Release.

[14] Mura S., Nicolas J., Couvreur P. (2013). Stimuli-responsive nanocarriers for drug delivery. Nat. Mater..

[15] Sulistio A., Lowenthal J., Blencowe A., Bongiovanni M.N., Ong L., Gras S.L., Zhang X., Qiao G.G. (2011). Folic acid conjugated amino acid-based star polymers for active targeting of cancer cells. Biomacromolecules.

[16] Yang H.-K., Bao J.-F., Mo L., Yang R.-M., Xu X.-D., Tang W.-J., Lin J.-T., Wang G.-H., Zhang L.-M., Jiang X.-Q. (2017). Bioreducible amphiphilic block copolymers based on PCL and glycopolypeptide as multifunctional theranostic nanocarriers for drug delivery and MR imaging. RSC Adv..

[17] Katsamakas S., Chatzisideri T., Thysiadis S., Sarli V. (2017). RGD-mediated delivery of small-molecule drugs. Future Med. Chem..

[18] Qian Z.M., Li H., Sun H., Ho K. (2002). Targeted drug delivery via the transferrin receptor-mediated endocytosis pathway. Pharmacol. Rev..

[19] Mould D.R., Meibohm B. (2016). Drug Development of Therapeutic Monoclonal Antibodies. BioDrugs.

[20] Zhao F., Yin H., Zhang Z., Li J. (2013). Folic acid modified cationic γ-cyclodextrin-oligoethylenimine star polymer with bioreducible disulfide linker for efficient targeted gene delivery. Biomacromolecules.

[21] Zhu D., Wu S., Hu C., Chen Z., Wang H., Fan F., Qin Y., Wang C., Sun H., Leng X. (2017). Folate-targeted polymersomes loaded with both paclitaxel and doxorubicin for the combination chemotherapy of hepatocellular carcinoma. Acta Biomater..

[22] Canal F., Vicent M.J., Pasut G., Schiavon O. (2010). Relevance of folic acid/polymer ratio in targeted PEG-epirubicin conjugates. J. Control Release.

[23] Chen Y., Liu W., Shang Y., Cao P., Cui J., Li Z., Yin X., Li Y. (2018). Folic acid-nanoscale gadolinium-porphyrin metal-organic frameworks: Fluorescence and magnetic resonance dual-modality imaging and photodynamic therapy in hepatocellular carcinoma. Int. J. Nanomed..

[24] Zhou Q., Zhang L., Yang T., Wu H. (2018). Stimuli-responsive polymeric micelles for drug delivery and cancer therapy. Int. J. Nanomed..

[25] Balendiran G.K., Dabur R., Fraser D. (2004). The role of glutathione in cancer. Cell Biochem. Funct..

[26] Zhang A., Zhang Z., Shi F., Ding J., Xiao C., Zhuang X., He C., Chen L., Chen X. (2013). Disulfide crosslinked PEGylated starch micelles as efficient intracellular drug delivery platforms. Soft Matter.

[27] Thambi T., Yoon H.Y., Kim K., Kwon I.C., Yoo C.K., Park J.H. (2011). Bioreducible block copolymers based on poly(ethylene glycol) and poly(γ-benzyl L-glutamate) for intracellular delivery of camptothecin. Bioconjug. Chem..

[28] England R.M., Moss J.I., Gunnarsson A., Parker J.S., Ashford M.B. (2020). Synthesis and Characterization of Dendrimer-Based Polysarcosine Star Polymers: Well-Defined, Versatile Platforms Designed for Drug-Delivery Applications. Biomacromolecules.

[29] Willner L., Jucknischke O., Richter D., Roovers J., Zhou L.L., Toporowski P.M., Fetters L.J., Huang J.S., Lin M.Y., Hadjichristidis N. (1994). Structural Investigation of Star Polymers in Solution by Small-Angle Neutron Scattering. Macromolecules.

[30] Gao H. (2012). Development of star polymers as unimolecular containers for nanomaterials. Macromol. Rapid Commun..

[31] Burke J., Donno R., d’Arcy R., Cartmell S., Tirelli N. (2017). The Effect of Branching (Star Architecture) on Poly(d,l-lactide) (PDLLA) Degradation and Drug Delivery. Biomacromolecules.

[32] Loftsson T., Brewster M.E. (2012). Cyclodextrins as functional excipients: Methods to enhance complexation efficiency. J. Pharm. Sci..

[33] Liao R., Lv P., Wang Q., Zheng J., Feng B., Yang B. (2017). Cyclodextrin-based biological stimuli-responsive carriers for smart and precision medicine. Biomater. Sci..

[34] Elgamouz A., Nassab C., Bihi A., Mohamad S.A.I., Almusafri A.H.S.A., Alharthi S.S., Abdulla S.A.E., Patole S.P. (2021). Encapsulation Capacity of β-Cyclodextrin Stabilized Silver Nanoparticles towards Creatinine Enhances the Colorimetric Sensing of Hydrogen Peroxide in Urine. Nanomaterials.

[35] Toomari Y., Namazi H., Akbar E.A. (2015). Synthesis of the dendritic type β-cyclodextrin on primary face via click reaction applicable as drug nanocarrier. Carbohydr. Polym..

[36] Toomari Y., Namazi H., Entezami A.A. (2015). Fabrication of biodendrimeric β-cyclodextrin via click reaction with potency of anticancer drug delivery agent. Int. J. Biol. Macromol..

[37] López-Méndez L.J., González-Méndez I., Aguayo-Ortiz R., Dominguez L., Alcaraz-Estrada S.L., Rojas-Aguirre Y., Guadarrama P. (2018). Synthesis of a poly(ester) dendritic β-cyclodextrin derivative by “click” chemistry: Combining the best of two worlds for complexation enhancement. Carbohydr. Polym..

[38] Gou P.F., Zhu W.P., Shen Z.Q. (2010). Synthesis, self-assembly, and drug-loading capacity of well-defined cyclodextrin-centered drug-conjugated amphiphilic A(14)B(7) Miktoarm star copolymers based on poly(epsilon-caprolactone) and poly(ethylene glycol). Biomacromolecules.

[39] Li N., Luo H.C., Ren M., Zhang L.M., Wang W., Pan C.L., Yang L.Q., Lao G.J., Deng J.J., Mai K.J. (2017). Efficiency and Safety of β-CD-(D(3))(7) as siRNA Carrier for Decreasing Matrix Metalloproteinase-9 Expression and Improving Wound Healing in Diabetic Rats. ACS Appl. Mater. Interfaces.

[40] Méndez-Ardoy A., Guilloteau N., Di Giorgio C., Vierling P., Santoyo-González F., Ortiz Mellet C., García Fernández J.M. (2011). β-Cyclodextrin-based polycationic amphiphilic “click” clusters: Effect of structural modifications in their DNA complexing and delivery properties. J. Org. Chem..

[41] Su H., Liu Y., Wang D., Wu C., Xia C., Gong Q., Song B., Ai H. (2013). Amphiphilic starlike dextran wrapped superparamagnetic iron oxide nanoparticle clsuters as effective magnetic resonance imaging probes. Biomaterials.

[42] Xu J., Liu S. (2009). Synthesis of well-defined 7-arm and 21-arm poly(N-isopropylacrylamide) star polymers with β-cyclodextrin cores via click chemistry and their thermal phase transition behavior in aqueous solution. J. Polym. Sci. Part A Polym. Chem..

[43] Schatz C., Louguet S., Le Meins J.-F., Lecommandoux S. (2009). Polysaccharide-block-polypeptide Copolymer Vesicles: Towards Synthetic Viral Capsids. Angew. Chem. Int. Ed..

[44] Le H.T., Jeon H.M., Lim C.W., Kim T.W. (2014). Synthesis, cytotoxicity, and phase-solubility study of cyclodextrin click clusters. J. Pharm. Sci..

[45] Chang L., Deng L., Wang W., Lv Z., Hu F., Dong A., Zhang J. (2012). Poly(ethyleneglycol)-b-poly(ε-caprolactone-co-γ-hydroxyl-ε- caprolactone) bearing pendant hydroxyl groups as nanocarriers for doxorubicin delivery. Biomacromolecules.

[46] Tang Y., Li Y., Xu R., Li S., Hu H., Xiao C., Wu H., Zhu L., Ming J., Chu Z. (2018). Self-assembly of folic acid dextran conjugates for cancer chemotherapy. Nanoscale.

[47] Wilhelm M., Zhao C.L., Wang Y., Xu R., Winnik M.A., Mura J.L., Riess G., Croucher M.D. (1991). Poly(styrene-ethylene oxide) block copolymer micelle formation in water: A fluorescence probe study. Macromolecules.

[48] Mi Y., Liu Y., Feng S.S. (2011). Formulation of Docetaxel by folic acid-conjugated d-α-tocopheryl polyethylene glycol succinate 2000 (Vitamin E TPGS(2k)) micelles for targeted and synergistic chemotherapy. Biomaterials.

[49] Yang X., Chen Y., Yuan R., Chen G., Blanco E., Gao J., Shuai X. (2008). Folate-encoded and Fe3O4-loaded polymeric micelles for dual targeting of cancer cells. Polymer.

[50] Sun H., Guo B., Li X., Cheng R., Meng F., Liu H., Zhong Z. (2010). Shell-sheddable micelles based on dextran-SS-poly(epsilon-caprolactone) diblock copolymer for efficient intracellular release of doxorubicin. Biomacromolecules.

[51] Zunino F., Di Marco A., Zaccara A., Gambetta R.A. (1980). The interaction of daunorubicin and doxorubicin with DNA and chromatin. Biochim. Biophys. Acta.

[52] Zunino F., Gambetta R., Di Marco A., Velcich A., Zaccara A., Quadrifoglio F., Crescenzi V. (1977). The interaction of adriamycin and its beta anomer with DNA. Biochim. Biophys. Acta.

[53] Shuai X., Ai H., Nasongkla N., Kim S., Gao J. (2004). Micellar carriers based on block copolymers of poly(epsilon-caprolactone) and poly(ethylene glycol) for doxorubicin delivery. J. Control Release.

[54] Yang X., Grailer J.J., Rowland I.J., Javadi A., Hurley S.A., Matson V.Z., Steeber D.A., Gong S. (2010). Multifunctional stable and pH-responsive polymer vesicles formed by heterofunctional triblock copolymer for targeted anticancer drug delivery and ultrasensitive MR imaging. ACS Nano.

[55] Sanson C., Diou O., Thévenot J., Ibarboure E., Soum A., Brûlet A., Miraux S., Thiaudière E., Tan S., Brisson A. (2011). Doxorubicin loaded magnetic polymersomes: Theranostic nanocarriers for MR imaging and magneto-chemotherapy. ACS Nano.

[56] Taktak S., Sosnovik D., Cima M.J., Weissleder R., Josephson L. (2007). Multiparameter magnetic relaxation switch assays. Anal. Chem..

